# Two Hits for Bone Regeneration in Aged Patients: Vertebral Bone Marrow Clot as a Biological Scaffold and Powerful Source of Mesenchymal Stem Cells

**DOI:** 10.3389/fbioe.2021.807679

**Published:** 2022-01-18

**Authors:** Francesca Salamanna, Deyanira Contartese, Veronica Borsari, Stefania Pagani, Giovanni Barbanti Brodano, Cristiana Griffoni, Alessandro Ricci, Alessandro Gasbarrini, Milena Fini

**Affiliations:** ^1^ Complex Structure Surgical Sciences and Technologies, IRCCS Istituto Ortopedico Rizzoli, Bologna, Italy; ^2^ Department of Oncological and Degenerative Spine Surgery, IRCCS Istituto Ortopedico Rizzoli, Bologna, Italy; ^3^ Anesthesia-Resuscitation and Intensive Care, IRCCS Istituto Ortopedico Rizzoli, Bologna, Italy

**Keywords:** clot, bone marrow, regenerative medicine, orthopedic injuries, aging

## Abstract

Recently, the use of a new formulation of bone marrow aspirate (BMA), the BMA clot, has been described. This product entails a naturally formed clot from the harvested bone marrow, which retains all the BMA components preserved in a matrix biologically molded by the clot. Even though its beneficial effects were demonstrated by some studies, the impact of aging and aging-associated processes on biological properties and the effect of BMA cell-based therapy are currently unknown. The purpose of our study was to compare selected parameters and properties of clotted BMA and BMA-derived mesenchymal stem cells (MSCs) from younger (<45 years) and older (>65 years) female donors. Clotted BMA growth factors (GFs) expression, MSCs morphology and viability, doubling time, surface marker expression, clonogenic potential, three-lineage differentiation, senescence-associated factors, and Klotho synthesis from younger and older donors were analyzed. Results indicated that donor age does not affect tissue-specific BMA clot regenerative properties such as GFs expression and MSCs morphology, viability, doubling time, surface antigens expression, colony-forming units, osteogenic and adipogenic differentiation, and Klotho and senescence-associated gene expression. Only few differences, i.e., increased platelet-derived growth factor-AB (PDGF-AB) synthesis and MSCs Aggrecan (ACAN) expression, were detected in younger donors in comparison with older ones. However, these differences do not interfere with all the other BMA clot biological properties. These results demonstrated that BMA clot can be applied easily, without any sample processing and avoiding potential contamination risks as well as losing cell viability, proliferation, and differentiation ability, for autologous transplantation in aged patients. The vertebral BMA clot showed two successful hits since it works as a biological scaffold and as a powerful source of mesenchymal stem cells, thus representing a novel and advanced therapeutic alternative for the treatment of orthopedic injuries.

## Introduction

Bone has well-documented natural healing properties which however change throughout life with aging (Boskey and Coleman, 2010; Curtis et al., 2015). Aging is accompanied by the increased incidence of bone diseases and reduced fracture-healing capacity, which require successful therapies able to enhance bone healing and regeneration (Boskey and Coleman, 2010; Curtis et al., 2015). In this context, the therapeutic potential of adult mesenchymal stem cells (MSCs) for bone repair has been long proposed and investigated (Shang et al., 2021). MSCs from several sources have been employed in the field of bone regeneration including bone marrow (BM), adipose tissue, umbilical cord, and dental-related tissues (Shang et al., 2021). BM-MSCs were the first identified and, to date, represent the most commonly used MSCs source for bone regeneration (Shang et al., 2021). These cells can be implanted after culture expansion or injected as whole bone marrow aspirate (BMA) or BM concentrate (BMC) (Shang et al., 2021). Considering regulation on *in vitro* cell processing, the use of cultured BM-MSCs in Europe and in the United States is restricted. Conversely, whole BMA or BMC involves minimal cell manipulation and can be used in clinical practice to treat bone diseases in a “*one-step*” procedure (Hernigou et al., 2005). Although only 0.01–0.001% of MSCs is found among the totality of mononuclear cells in BMA, the concurrent presence of nonadherent osteogenic cells and the establishment of cell–cell interactions suggest that the use of whole BMA, instead of BMC or expanded and purified MSCs, is preferable for bone cell therapy (Hernigou et al., 2005). Recently, our research group also demonstrated that MSCs derived from human clotted BMA have higher growth kinetics in comparison to MSCs derived from human un-clotted BMA (whole and concentrate) as well as higher growth factors expression (transforming growth factor beta, TGF-β; vascular-endothelial growth factor, VEGF-A; fibroblast growth factor 2, FGF2) and higher ability to differentiate toward the osteogenic and chondrogenic phenotype (Salamanna et al., 2020). These data were also confirmed by an *in vivo* study by Lim and colleagues that underlined that the therapeutic potential of autologous bone graft and BMA clot in the repair of ulnar defects are similar, with the advantage of BMA clot being associated with a lower risk of complications (Lim et al., 2019). A recent literature review further elucidates the characteristics found in BMA clot and its application in the clinical scenario (Santos Duarte Lana et al., 2020). In this context, to confirm and strengthen data on the clinical use of BMA clot for bone regeneration, a pilot clinical study on clotted vertebral BMA is ongoing at our institution (Protocol: CVOD. coagulo_CE-AVEC 587/2020/Sper/IORS). Although BMA clot has key properties and characteristics that make it a promising cell therapy strategy for bone repair and regeneration, aging could be a critical point as aging-associated processes could impact on the biological properties and clinical efficacy of BMA clot (D’Ippolito et al., 1999). During aging, resident MSCs inside the BM are affected by both intrinsic and extrinsic factors, which alter their functions (D’Ippolito et al., 1999). Aging effects on BM-MSC properties, such as telomere length, cell proliferation, differentiation ability, epigenetics, and secretome, have been discussed and demonstrated in several preclinical studies and systematic reviews (Boyette and Tuan, 2014; Baker et al., 2015; Charif et al., 2017; Ganguly et al., 2017). Aging also decreases the expression level of FGF2 which in turn leads to the reduction of BM-MSCs proliferative capacity (Gibon et al., 2016). Additionally, aging reduces the density of MSCs in BM and compromises their osteogenic potential (D’Ippolito et al., 1999). Interestingly, a study by D’Ippolito et al. showed that in human vertebral BM, the number of MSCs with osteogenic potential decreases early during aging, which may be responsible for the age-related reduction in bone formation, mechanical properties, and bone integrity (D’Ippolito et al., 1999). Increasing evidence also suggests that the aging BM-MSCs promote adipogenesis at the expense of osteogenesis, thus resulting in decreased bone formation capacity (Marędziak et al., 2016). The secretion of senescence-associated secretory phenotype (SASP) products from senescent MSCs in BM has also been evidenced during aging (Turinetto et al., 2016; Ma et al., 2018; Malaise et al., 2019). Interleukin-6 (IL-6), one of the most critical SASP factors, has been shown to drive osteoclastogenesis and negatively regulate osteoblast differentiation (Turinetto et al., 2016; Ma et al., 2018). The knockdown of IL-6 significantly enhanced runt-related transcription factor 2 (Runx2) and collagen type I alpha 1 chain (COL1A1) expression in osteoblasts while decreasing the expression of osteoclast-related genes such as tartrate-resistant alkaline phosphatase (TRAP), metalloproteinase (MMP)-9, and cathepsin K (CTSK) (Turinetto et al., 2016; Ma et al., 2018; Malaise et al., 2019). Furthermore, a contribution to MSCs senescence is also given by the Klotho gene, a gene that encodes Klotho antiaging protein (Kuro-o, 2012). Loss-of-function mutation of Klotho in mice leads to a syndrome resembling human premature aging, including defects in skeletal mineralization and osteoporosis (Kuro-o, 2012) *scenario*. These age-related factors could limit the use of BM stem cell as cell-based therapy in the clinical scenario.

Considering that MSCs from clotted vertebral BMA showed better biological properties in comparison to the whole and concentrated BM, it represents a point-of-care orthobiologic product that uniquely and simultaneously delivers growth factors and MSCs (Salamanna et al., 2020). The goal of this study was to evaluate the influence of donor age on human vertebral clotted BMA, one of the skeletal sites with the highest bone turnover, characterizing and comparing the biological properties of MSCs from younger (<45 years) and older female donors (>65 years).

## Materials and Methods

### Bone Marrow Clot Collection

**FIGURE 1:**
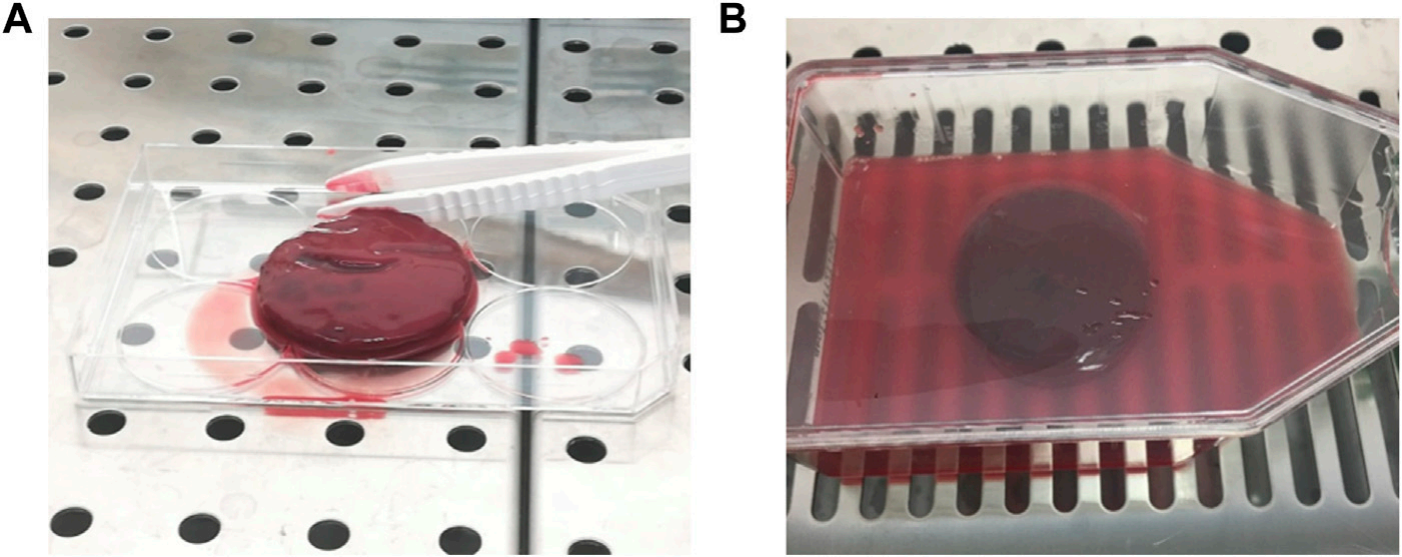
Clotted BMA **(A)** after the remotion from the disposable plastic graduated container and **(B)** in the culture flask.

The study, which is part of an ongoing pilot clinical study on the efficacy of clotted vertebral BMA in spinal fusion surgery, was approved by the Local Ethics Committee of the Emilia Romagna Region (Comitato Etico Indipendente Area Vasta Emilia Centro, Protocol n. CE-AVEC 587/2020/Sper/IOR S) and was carried out in accordance with relevant guidelines and regulations (IRCCS Istituto Ortopedico Rizzoli has kept the ISO 9001 Quality certification since 2008). Written informed consent was obtained from all subjects involved in the study. Inclusion criteria were patients with degenerative spinal diseases submitted to multilevel instrumented arthrodesis (minimum 2–maximum 6 levels), patients of legal age, and patients able to give their consent and answer self-administered questionnaires. Exclusion criteria were human immunodeficiency virus (HIV), hepatitis B virus (HBV), hepatitis C virus (HCV), diabetes, pregnancy, bone diseases, drugs active on bone metabolism, primary bone tumors, metastases, minors, and/or patients incapable of giving consent personally. Human BMA was harvested from vertebral pedicles of six female patients, 3 younger (mean age: 44 ± 0.5) and 3 older (mean age: 68.6 ± 2.5) (body mass index and bone mineral index matched), undergoing spinal surgical procedures involving posterolateral arthrodesis. None of the patients had a diagnosis of osteoporosis. Eight milliliters (1 or 2 ml from each vertebra depending on the length of the arthrodesis) of human vertebral BMA were harvested into a 10-ml syringe during the preparation of the pilot hole for pedicle screw fixation. Subsequently, BMA was divided into two equal parts of 4 ml each in a sterile disposable container without any anticoagulant and immediately transferred to the laboratory. BMA without anticoagulant clotted in about 15–30 min and was transferred in culture flasks with Dulbecco’s modified Eagles medium (DMEM, Sigma–Aldrich, St. Louis, MO), containing 10% fetal bovine serum (FBS, Lonza), 100 U/ml penicillin, 100 μg/ml streptomycin (Gibco, Life Technologies, Carlsbad, CA), and 5 μg/ml plasmocin (Invivogen, San Diego, CA) (Figure 1). The cultures were incubated at 37°C in 5% CO_2_ and under hypoxia (2% O_2_), to better mimic the microenvironment of the stem cells’ niche. Culture media was changed every three days.

### Cell Morphology and Viability

After removing nonadherent cells (after 72 h, when the clot was almost completely dissolved), younger and older MSCs from clotted BMAs were observed twice a week up to 85–90% of cell confluence, and the images acquired by a standard light microscope (Nikon Eclipse, Ti-U, Nikon Italia Srl, Italy) equipped with a digital camera at 4× and 10× magnification. At confluence, cell viability was assessed via LIVE/DEAD^®^ assay (Thermo Fisher Scientific, Waltham, MA, United States) according to the manufacturer’s instructions. Briefly, LIVE/DEAD staining was performed by incubating the cells with a working solution of calcein-AM (4 μM) and ethidium homodimer-1 (2 μM) for 45 min at 37°C. Cells were washed with phosphate-buffered saline (PBS), and images acquired by a fluorescence microscope (Nikon Eclipse, Ti-U, Nikon Italia Srl, Italy) equipped with a digital camera at 10× and 20× magnification. The green and red fluorescence was typical of live and dead cells, respectively.

### Immunoenzymatic Growth Factors Assay

Cells were plated at a density of 7×10^3^ cells/cm^2^ in 12-well plates. Each sample was tested in triplicate. At 72 h, media from younger and older clotted BMAs were collected and centrifuged to remove particulates. Aliquots of supernatant were dispensed in Eppendorf tubes for storage at −20 C and subsequently assayed with the following immunoenzymatic kits: basic fibroblast growth factor (bFGF), VEGF, TGF-β 1, bone morphogenetic protein 2 (BMP-2), platelet-derived growth factor AB (PDGF-AB), platelet-derived growth factor C (PDGF-C) (Sigma–Aldrich, St. Louis, MO).

### Immunophenotyping

At passage 0, the characteristics of younger and older MSCs from clotted BMAs were investigated by characterizing cell-surface markers of isolated cells using flow cytometry analysis. To test surface antigen expression, 0.5–1 × 10^5^ MSCs for each antigen was washed with PBS, centrifuged at 260 g for 5 min, and incubated at 4 C for 30 min in flow cytometry buffer (FCB, 2% FBS in PBS) adding 0.5 μg/ml of fluorescein isothiocyanate (FITC)-conjugated antibody against CD31, CD45, CD34, CD44, CD73, CD90, and CD105. FITC-conjugated nonspecific immunoglobulin G (IgG) was used as isotype control (BioLegend, San Diego, CA, United States). Cell fluorescence was evaluated with FACSCanto II instrument (Becton Dickinson, Franklin Lakes, NJ, United States) and analyzed by FACSDiva software (Becton Dickinson).

### Population Doubling Time

To compare the growth characteristics of younger and older MSCs derived from clotted BMA, the population doubling time (PDT) (period required for cells to proliferate or grow) was measured (in triplicate) at passage 1. Cells were plated at a density of 7 × 10^3^ cells/cm^2^ in 12-well plates. Each sample was tested in triplicate. On the 10th day, the cells were harvested and counted by erythrosine vital dye. CPDs (cumulative population doublings) and PDT were calculated by using the following formulae:

$$CPD_{S}=\log\left(N/N0\right)\times 3.31,$$

$$\mathrm{PDT=CT/CP}D_{s},$$

where N is the final number of cells, N0 is the initial number of cells seeded, and CT is the time in culture.

### Colony-Forming Units

At passage 1, two hundred MSCs/cm^2^ from younger and older clotted BMAs were plated onto 12-well plates and cultured for 10 days to evaluate the number of colony-forming units. Each sample was tested in triplicate. At the endpoint, cells were fixed with 10% formalin for 20 min and stained with 0.1% toluidine blue in 1% paraformaldehyde (PFA) for 1 h. The aggregates with ≥20 cells were visually scored as colonies and counted with a light microscope (Olympus BX51).

### Trilineage Differentiation (Osteo-, Adipo-, and Chondrogenesis)

#### Culture

At passage 1, osteogenic, adipogenic, and chondrogenic differentiations were induced for younger and older MSCs from clotted BMAs. Each sample was tested in triplicate. To induce osteogenic and adipogenic differentiation, MSCs were plated at a density of 7.0 × 10^3^ cells per cm^2^ onto 12-well plates and incubated in a culture medium (DMEM with 10% FBS, 100 U/ml penicillin, 100 μg/ml streptomycin, and 5 μg/ml plasmocin) for 24 h. After 24 h, the medium was replaced with osteogenic and adipogenic medium, and MSCs were cultured for 15 days. Osteogenic medium consisted of culture medium supplemented with dexamethasone 10^−8^ M, ascorbic acid 50 μg/ml, and β-glycerophosphate 10 mM while adipogenic medium consisted of culture medium supplemented with isobutylmethylxanthine 500 μM, indomethacin 100 μM, dexamethasone 1 × 10^−6^, and insulin 10 μg/ml. Osteogenic or adipogenic induction medium was changed every three days for 15 days.

To induce chondrogenesis, 2.5 × 10^5^ cells per tube were pelleted (as micromasses) at 260 g for 20 min. Pellet cultures were incubated for 24 h in culture medium before being switched to chondrogenic medium and grown for further 30 days. Chondrogenic medium was changed every three days for 30 days and consisted of culture medium supplemented with 5 μg/ml insulin, 5 μg/ml transferrin and 5 μg/ml selenous acid, 0.1 μM dexamethasone, 0.17 mM ascorbic acid–2-phosphate, 1 mM sodium pyruvate, 0.35 mM proline, and 10 μg/ml transforming growth factor-β3 (TGF-β3).

#### Staining

In order to evaluate differentiation potentials, after 15 days, osteogenic cultures were fixed in 10% formaldehyde for 15 min and stained with 2% Alizarin Red S (Sigma-Aldrich) for 30 min at room temperature to detect calcium deposits. To detect lipid accumulation after 15 days, adipogenic cultures were fixed in 4% PFA for 10 min at room temperature and stained with 1.8% Oil red O (Sigma-Aldrich) for 15 min at room temperature. LIVE/DEAD staining was also used to assess cell viability after 15 days of culture in both osteogenic and adipogenic medium as previously reported. After 30 days, chondrogenic micromasses were fixed in 10% formaldehyde for 30 min, washed in distilled water, and dehydrated in increasing ethanol series. Finally, they were clarified in xylene (VWR International, Milan, Italy) and embedded in paraffin (Thermo Fisher Scientific, Waltham, MA) blocks. Blocks were sectioned along a transversal plane and cut into 5 μm sections. Three consecutive sections for each sample were stained with Alcian blue–nuclear fast red. Histological images were taken with a digital pathology slide scanner (Aperio-ScanScope, Leica Biosystems) and evaluated at 4×, 10×, 20×, and 80× magnification.

#### Gene Expression

Total RNA was extracted from BMA clot-derived MSCs which differentiated toward osteogenic, adipogenic, chondrogenic lineages. RNA extraction was carried out using the commercial RNeasy Mini Kit (Purelink™ RNA Mini Kit, Ambion by Life Technologies, Carlsbad, CA, United States), and then, it was quantified by a NANODROP spectrophotometer (NANODROP 2720, Thermal Cycler, Applied Biosystem), reverse transcribed using the Superscript Vilo cDNA synthesis kit (Life Technologies), and diluted to the final concentration of 5 ng/μL. 10 ng of cDNA was tested in duplicate for each sample. Gene expression was evaluated by semiquantitative PCR analysis, using the SYBR green PCR master mix (QIAGEN GmbH, Hilden, Germany), in a LightCycler 2.0 Instrument (Roche Diagnostics, GmbH, Manheim, Germany). Primer details for all genes analyzed are reported in Table 1. Each sample was tested in triplicate. The protocol included a denaturation cycle at 95°C for 15 min, 25 to 40 cycles of amplification (95°C for 15 s, appropriate annealing temperature for each target gene for 20 s and 72°C for 20 s) (in Table 1 the primers’ details), and a melting curve analysis to check for amplicon specificity. The mean threshold cycle was determined for each sample and used for the calculation of relative expression using the Livak method (2^−ΔCt^), with GAPDH as the reference gene (Schmittgen and Livak, 2008).

**TABLE 1:** Primer details for osteo-, adipo-, and chondrogenic gene.

Gene	Primer forward	Primer reverse	Amplicon lenght	Annealing temperature
GAPDH	5′-TGG​TAT​CGT​GGA​AGG​ACT​CA-3′	5′-GCA​GGG​ATG​ATG​TTC​TGG​A -3′	123 bp	56°C
ACAN	5′-TCG​AGG​ACA​GCG​AGG​CC-3′	5′-TCG​AGG​GTG​TAG​CGT​GTA​GAG​A-3′	85 bp	60°C
SOX 9	5′-GAG​CAG​ACG​CAC​ATC​TC-3′	5′-CCT​GGG​ATT​GCC​CCG​A-3′	118 bp	60°C
COL2A1	QuantiTect Primer Assay (Qiagen) Hs_COL2A1_1_SG		94 bp	55°C
COL1A1	QuantiTect Primer Assay (Qiagen) Hs_COL1A1_1_SG		118 bp	55°C′
BGLAP	QuantiTect Primer Assay (Qiagen) Hs_BGLAP_1_SG		90 bp	55°C′
*RUNX2*	QuantiTect Primer Assay (Qiagen) Hs_RUNX2_1_SG		101 bp	55°C′
PPARg	QuantiTect Primer Assay (Qiagen) Hs_PPARG_1_SG		113 bp	55°C′
ADIPOQ	QuantiTect Primer Assay (Qiagen) Hs_ADIPOQ_1_SG		140 bp	55°C′

### Klotho and Senescence-Associated Gene Expression

At passage 1, 7 × 10^3^ MSCs/cm^2^ from younger and older BMAs’ clot were plated onto 12-well plates and cultured with culture medium (DMEM with 10% FBS, 100 U/ml penicillin, 100 μg/ml streptomycin, and 5 μg/ml plasmocin) for 10 days to evaluate senescence-associated gene expression as previously described for osteogenic, adipogenic, chondrogenic differentiation. Primer details for all genes analyzed are reported in Table 2.

**TABLE 2:** Primer details for the senescence-associated gene.

Gene	Primer forward	Primer reverse	Amplicon lenght	Annealing temperature
GAPDH	5′-TGG​TAT​CGT​GGA​AGG​ACT​CA-3′	5′-GCA​GGG​ATG​ATG​TTC​TGG​A -3′	123 bp	56°C
IL8	5′-ATG​ACT​TCC​AAG​CTG​GCC​GTG-3′	5′-TTA​TGA​ATT​CTC​AGC​CCT​CTT​CAA​AAA​CTT​CTC-3′	300 bp	60°C
IL1A	QuantiTect Primer Assay (Qiagen) Hs_IL1A_1_SG		74 bp	55°C′
IL 1B	QuantiTect Primer Assay (Qiagen) Hs_IL1B_1_SG		117 bp	55°C′
IL 6	QuantiTect Primer Assay (Qiagen) Hs_IL6_1_SG		107 bp	55°C′
CCL4L1 (MIP-1β)	QuantiTect Primer Assay (Qiagen) Hs_CCL4L1_4_SG		113 bp	55°C′
CXCL2 (MIP-2α)	QuantiTect Primer Assay (Qiagen) Hs_CXCL2_1_SG		110 bp	55°C′
KL (Klotho)	QuantiTect Primer Assay (Qiagen) Hs_KL_1_SG		143 bp	55°C′
TNFα	QuantiTect Primer Assay (Qiagen) Hs_TNF_1_SG		104 bp	55°C′
CCL2 (MCP-1)	QuantiTect Primer Assay (Qiagen) Hs_CCL2_1_SG		60 bp	55°C′

### Statistical Analysis

Statistical analysis was performed using the IBM^®^ SPSS^®^ Statistics v.23 software. Data are reported as mean ± standard deviation (SD) at a significant level of *p* < 0.05. After having verified normal distribution and homogeneity of variance (Levene test), data were analyzed with t-test to compare younger and older BMAs clot.

## Results

### Cell Morphology and Viability

**FIGURE 2:**
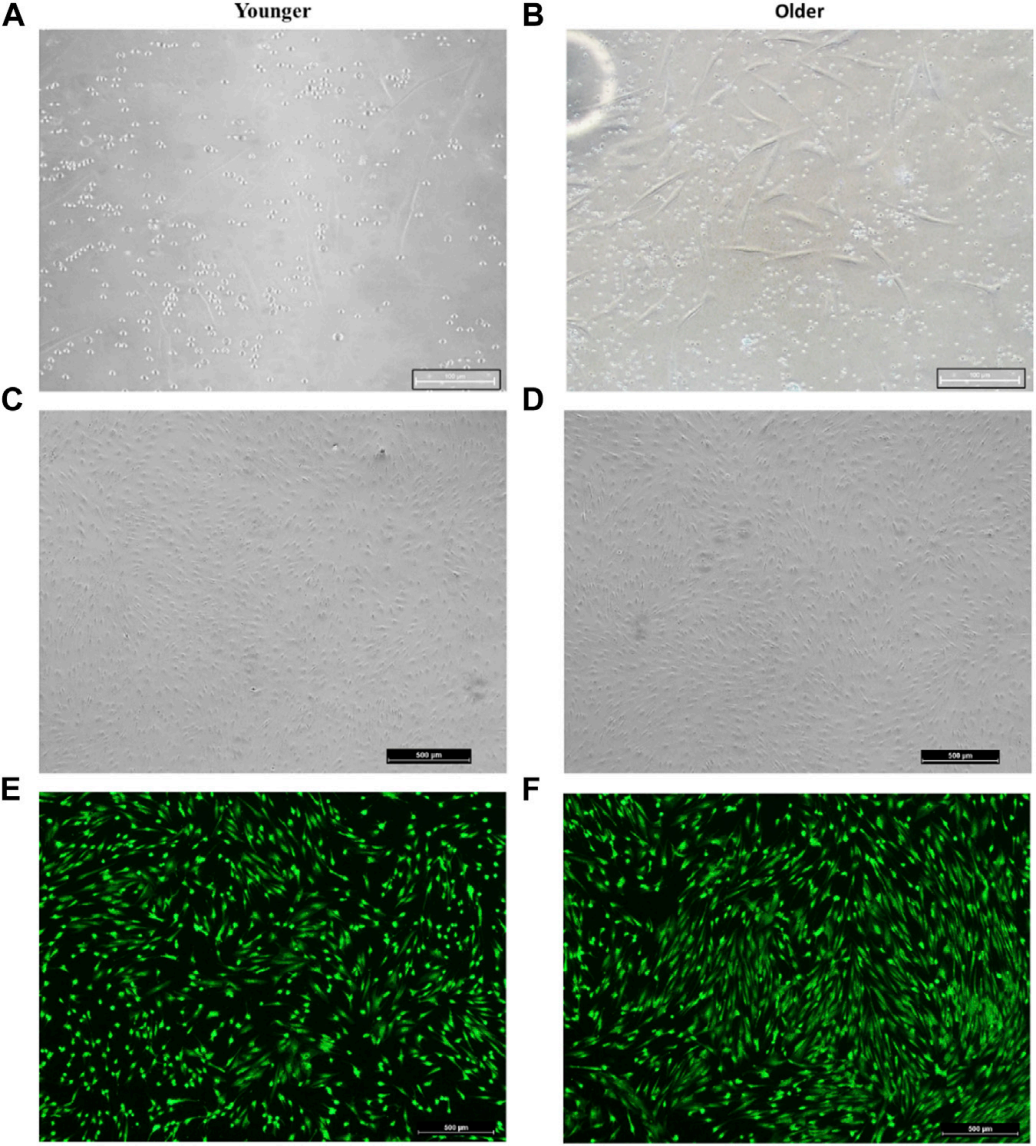
Representative image of **(A,C,E)** younger and **(B,D,F)** older MSCs from clotted BMA. **(A,B)** MSCs from clotted BMA at 6 days of culture; the presence of some red blood cells, platelets, and leucocytes. Magnification ×20. **(C,D)** MSCs from clotted BMA at 20 days of culture; the homogenous population of spindle-shaped and plastic-adherent cells. Magnification ×4. **(E,F)** Live/Dead analyses at 20 days of culture; green staining: viable cells; red staining: dead cells.

For both younger and older MSCs from clotted BMA, some red blood cells, platelets, and leucocytes were still observable within the first week of culture (Figures 2A,B). However, after 14 days of culture, most of the nonadherent cells were discarded, and at 20/25 days of culture, a homogenous population of spindle-shaped and plastic-adherent cells was observed in both MSCs samples (Figures 2C,D). The Live/Dead assay showed that all MSCs from clotted BMAs, both from younger and older donors, displayed excellent cellular viability (Figures 2E,F) without the presence of dead cells (in red staining).

### Immunoenzymatic GFs Assay

**FIGURE 3:**
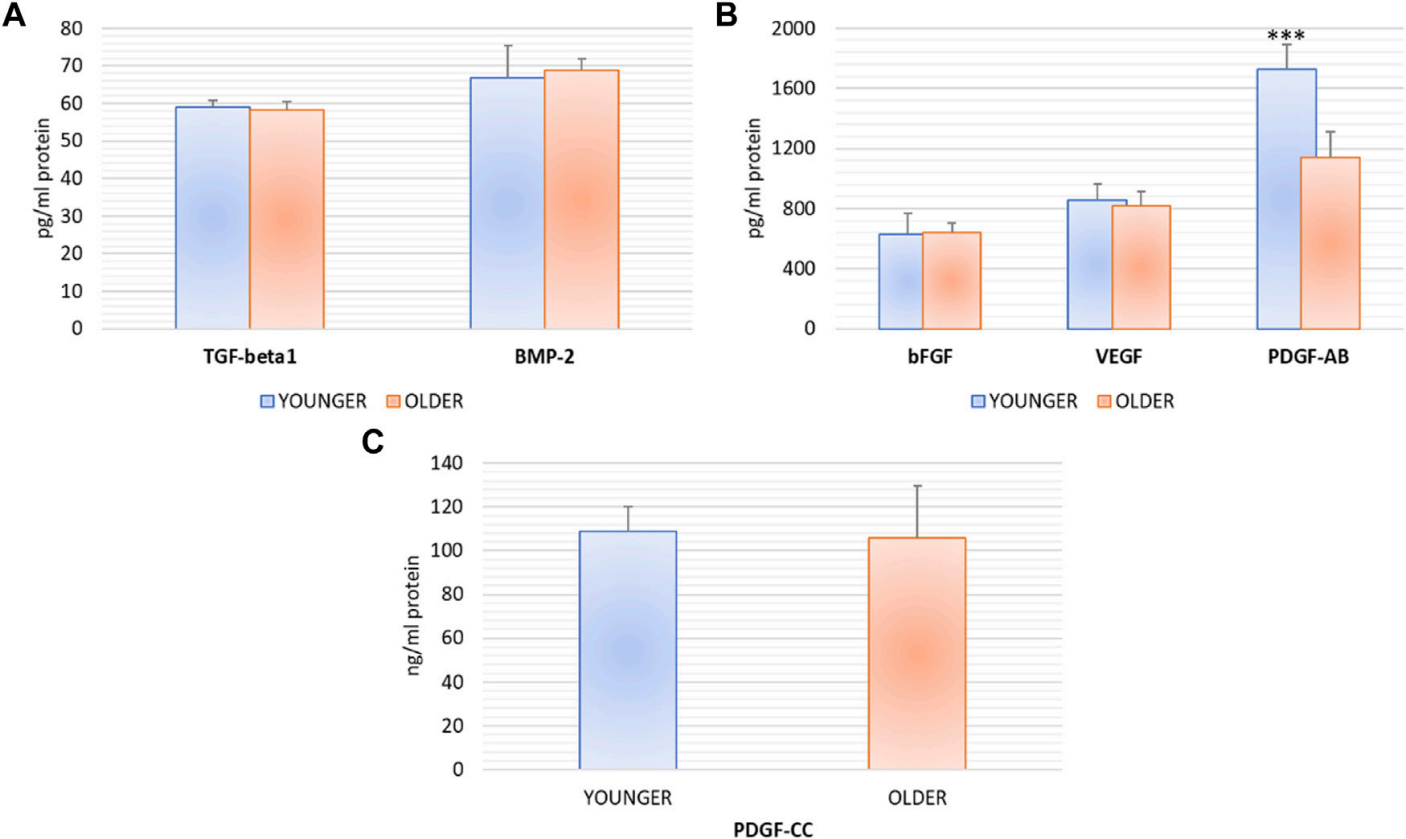
TGF-β 1, BMP-2, bFGF, VEGF, PDGF-AB, PDGF-CC protein synthesis after 72 h of the culture of BMAs’ clot from younger and older donors. PDGF-AB: *** younger versus older (***p < 0.0005).

At 72 h, supernatant from younger and older clotted BMAs did not show any significant difference for TGF-β 1 (*p* = 0.474), BMP-2 (*p* = 0.616), bFGF (*p* = 0.782), VEGF (*p* = 0.482), and PDGF-CC (*p* = 0.765) synthesis. Significant higher value of PDGF-AB was observed in BMAs from younger *versus* older donors (*p* < 0.0005) (Figure 3).

### Population Doubling Time

**FIGURE 4:**
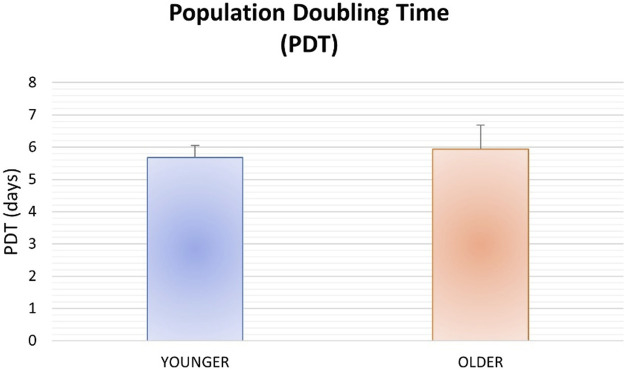
Population doubling time for MSCs from BMAs clot for younger and older donors.

No significant differences were found between younger and older MSCs from clotted BMAs for population doubling time (*p* = 0.357) (Figure 4).

### Immunophenotyping

**FIGURE 5:**
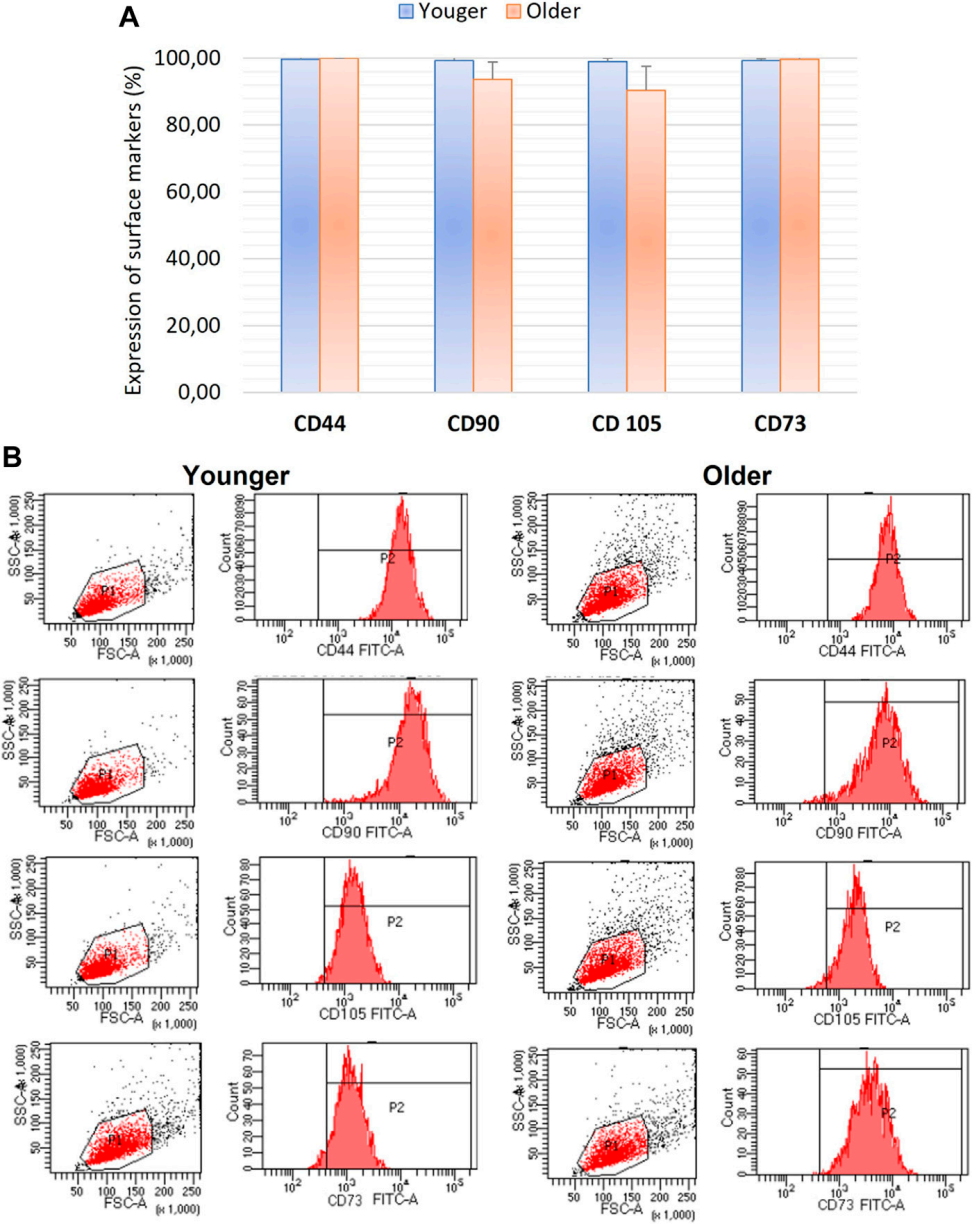
**(A)** Comparison of MSCs’ surface markers expression CD44, CD90, CD105, and CD73 for BMAs clot from younger and older donors; **(B)** representative histogram of the flow cytometry analysis (FACS) of the MSC-related CD surface markers in BMAs clot from younger and older donors. **(A)** and **(B)** demonstrated high positive expression of CD44, CD90, CD105, and CD73 in both MSCs from BMAs clot from younger and older donors.

**FIGURE 6:**
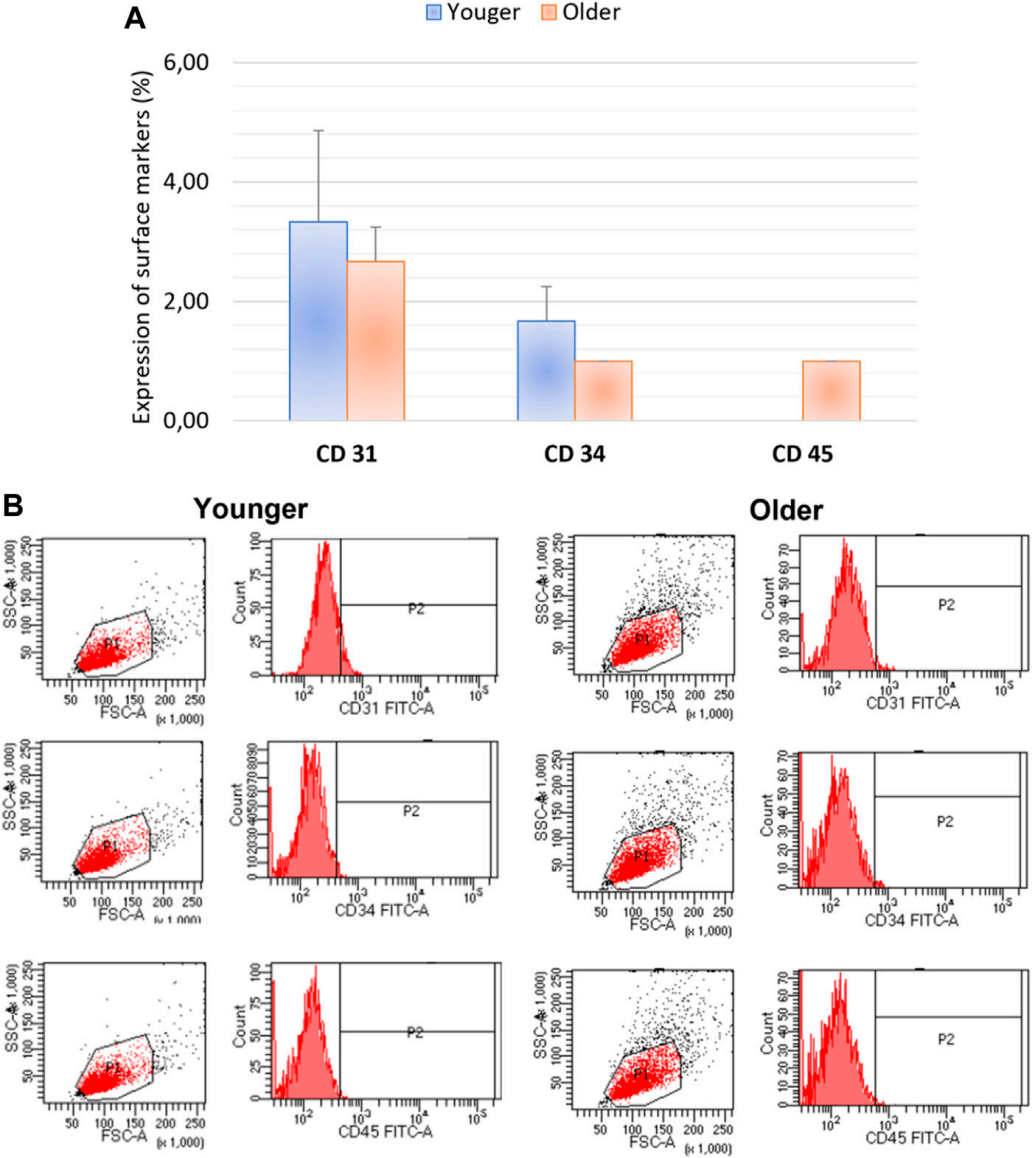
**(A)** Comparison of MSCs’ surface markers expression CD31, CD34, CD45 for BMAs clot from younger and older donors; **(B)** representative histogram of the flow cytometry analysis (FACS) of the MSC-related CD surface markers in BMAs clot from younger and older donors. **(A,B)** demonstrated negative expression of CD31, CD34, CD45 in both MSCs from BMAs clot from younger and older donors.

We did not observe any significant differences for CD44, CD90, CD105, and CD73 as well as CD31, CD34, CD45 expression between MSCs from younger and older clotted BMAs (Figures 5, 6).

### Colony-Forming Units

**FIGURE 7:**
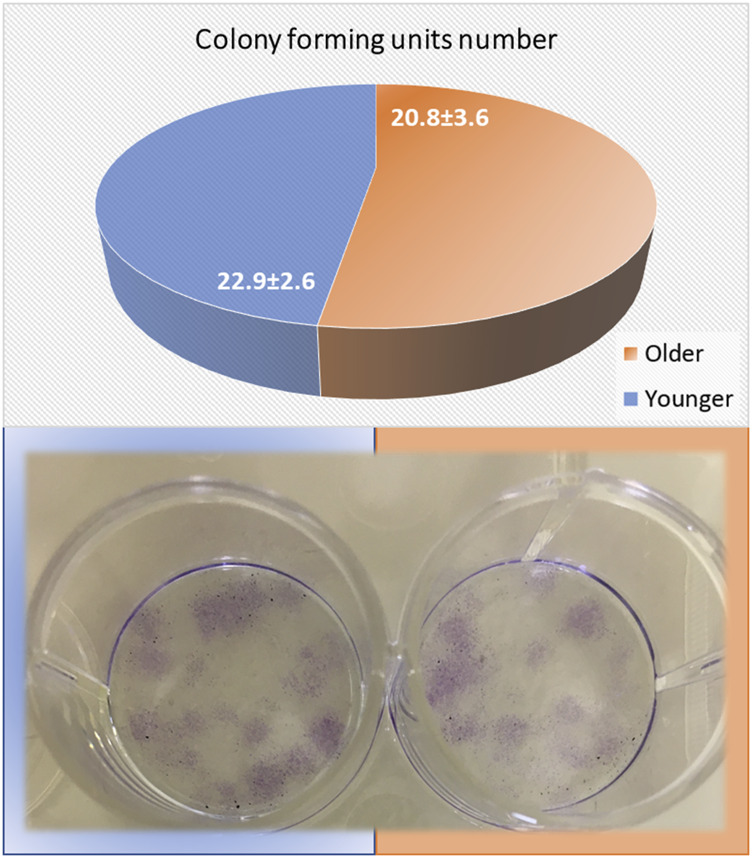
Colony-forming units (CFUs) count of MSCs from BMAs’ clot from younger and older donors after 10 days of culture in basal medium. The circle chart indicates the number of positive colonies. Representative images of CFUs onto 12-well plates were observed after 10 days of culture.

As seen in Figure 7, no significant differences were found between younger and older MSCs from clotted BMAs for the number of colony-forming units (*p* = 0.183).

### Trilineage Differentiation (Osteo-, Adipo-, and Chondrogenesis)

#### Staining

**FIGURE 8:**
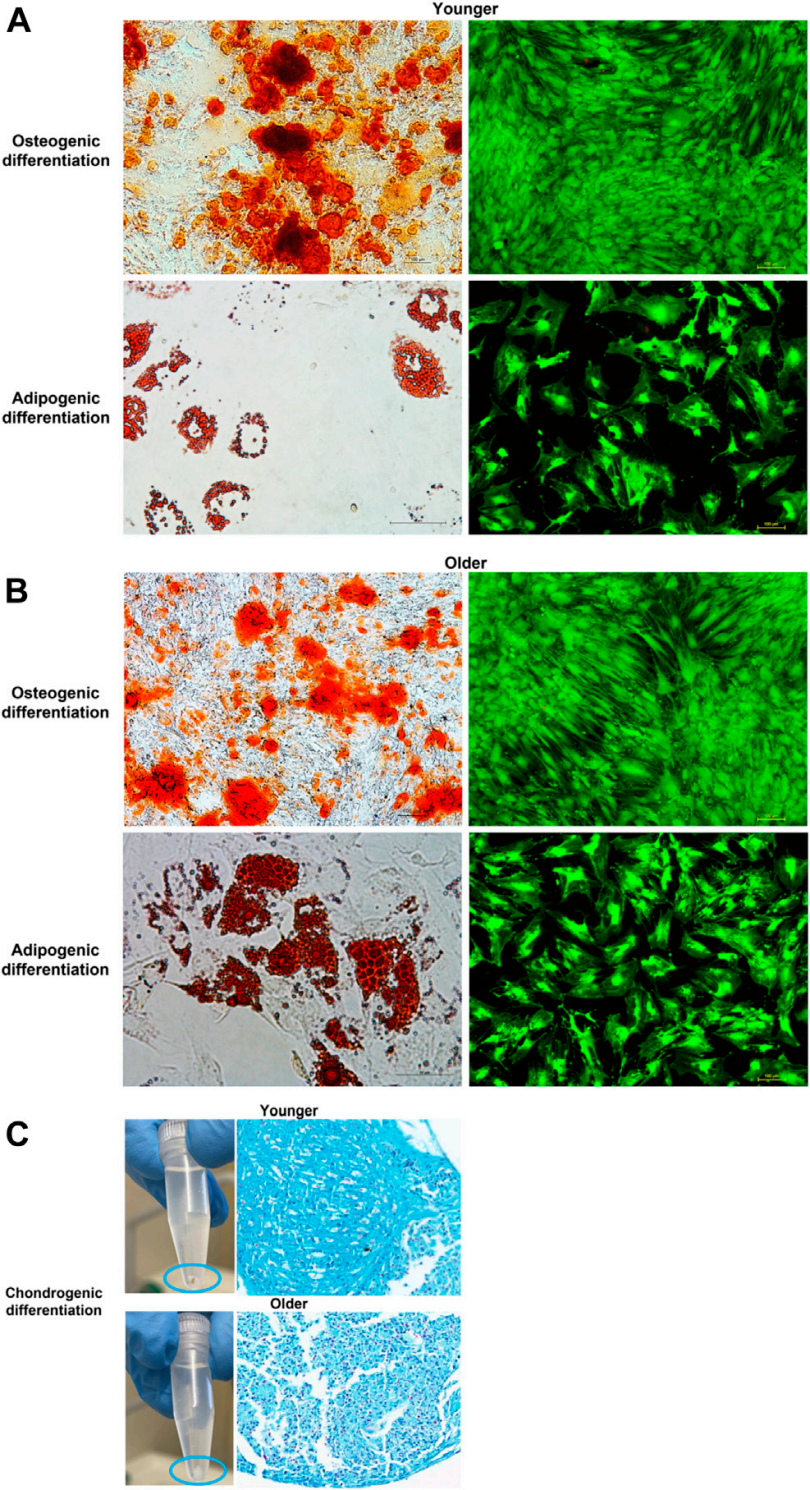
**(A)** Representative images of osteogenic and adipogenic differentiation of MSCs derived from clotted BMAs from younger donors (left: Alizarin Red S staining, magnification ×10, and Oil red O, magnification ×20; right: LIVE/DEAD fluorescent staining, magnification ×10), **(B)** from older donors (left: Alizarin Red S staining, magnification ×10 and, Oil red O, magnification ×20; right: LIVE/DEAD fluorescent staining, magnification ×10), and **(C)** images of chondrogenic differentiation from both younger and older donors (Alcian blue–nuclear fast red staining, magnification ×10) differentiation of MSCs derived from clotted BMAs from both younger and older donors.

Alizarin Red staining demonstrated the presence of mineralized (calcified) matrix areas in MSCs derived from clotted BMAs from both donors (Figures 8A,B). A massive mineralization with the deposition of inorganic calcium salts was observed for both samples (Figures 8A,B). Concerning the adipogenic differentiation, the Oil red O staining showed the presence of lipids droplets in MSCs derived from clotted BMAs from both donors. However, a decrease in cell density was observed when MSCs from clotted BMAs of younger donors were exposed to adipogenic medium (Figure 8A). For both samples, the viability of MSCs exposed to the osteogenic and adipogenic medium was confirmed by LIVE/DEAD staining (Figures 8A,B). In relation to the chondrogenic differentiation of MSCs derived from clotted BMAs from both donors, the presence of chondrocytes inside the lacunae organized in an isogenous group and separated by extracellular matrix was seen (Figure 8C).

#### Gene Expression

**FIGURE 9:**
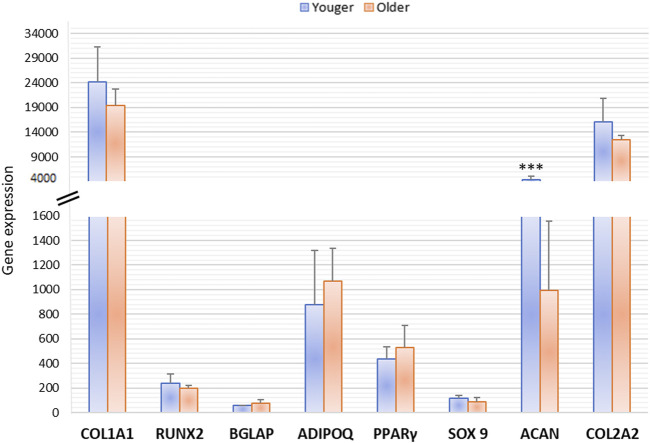
Gene expression measured by semiquantitative PCR of osteogenic (COL1A1, RUNX2, BGLAP), adipogenic (ADIPO Q, PPARγ), and chondrogenic (SOX9, ACAN, COL2A1) markers comparing the differentiation potential of MSCs derived from clotted BMAs from younger and older donors. ACAN: *** younger *versus* older (***p < 0.0005).

After 15 days of osteogenic and adipogenic differentiation, RT-PCR demonstrated no significant differences between MSCs from clotted BMAs from younger and older donors. A significant difference was detected only for Aggrecan (ACAN) expression that showed significant higher values in younger donors in comparison to older ones (*p * <  0.0005) (Figure 9).

## Klotho and Senescence-Associated Gene Expression

**FIGURE 10:**
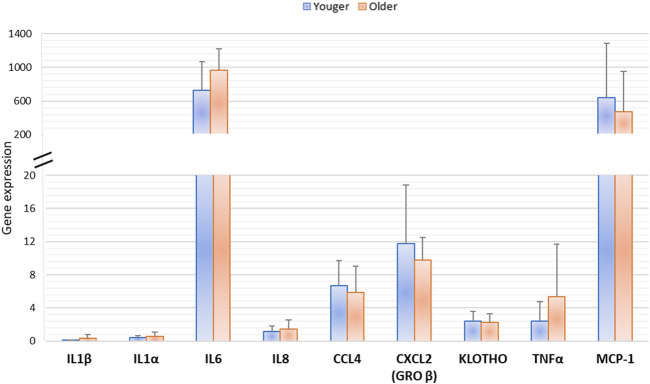
Gene expression of MSCs derived from clotted BMAs from younger and older donors measured by semiquantitative PCR for Klotho and senescence-associated gene expression (IL1β, IL1α, IL6, IL8, CCL4, CXCL2, TNFα, and MCP-1).

At 10 days of MSCs culture, Klotho and senescence-associated gene (IL1β, IL1α, IL6, IL8, CCL4, CXCL2, TNFα, and CCL-2) expression evaluated by RT-PCR showed no significant difference among vertebral BMA from younger and older donors (Figure 10).

## Discussion

The development of new and advanced therapeutic alternatives for bone healing and regeneration such as orthobiologics application is conveying optimism among orthopedic surgeons and researchers, as evidenced by the numerous studies and systematic reviews found in the literature (Alonso-Goulart et al., 2021; Zhang S et al., 2021; Dzobo, 2021; Fu et al., 2021; Wilson et al., 2021). Although several autologous BMA-based products, such as BMC and whole BMA, have been widely proposed for bone regeneration, the distinctive and enhanced biological and physical properties of clotted BMA make it an excellent candidate for autologous cell therapy (Veronesi et al., 2013). This product consists of a naturally formed clot from the harvested BMA, which retains all the BMA components preserved in a biological matrix by the clot. The formation of a BMA clot comprises the degranulation of platelets that deliver various osteotropic cytokines and GFs, such as PDGF-AB, FGF, and BMPs, as well as angiogenic factors derived by the fibrinolytic activity occurring within the clot (Muschler et al., 2003; Palta et al., 2014). Recent studies demonstrated that MSCs from the clotted BMA showed enhanced biological properties in comparison to concentrate and whole BM and that the clotted BMA is an effective and efficient alternative option to autograft for *in vivo* long bone healing (Lim et al., 2019; Salamanna et al., 2020). Thanks to its properties, the BMA clot behaves like an early hematoma, able not only to deliver new cells to the injury site but also to trigger an immunomodulatory cascade capable of attracting peripheral MSCs to the injury site. Nevertheless, to date, no studies evaluated the potential effects that aging could have on BMA clot biological properties (Santos Duarte Lana et al., 2020). Since aging is accompanied by the increased incidence of bone diseases and reduced fracture-healing capacity, it is of key importance to evaluate orthobiologics’ properties in the aging population. Much like other BMA-based products, the clotted BMA and the BMA-derived MSCs could be affected by both intrinsic and extrinsic alteration linked to aging that could potentially minimize its superior biological properties. The results of this study indicate for the first time that donor age does not affect tissue-specific BMA clot regenerative properties such as GFs expression (TGF-β1, BMP-2, bFGF, VEGF, and PDGF-CC) and MSCs morphology, viability, dynamics clone development (cell doubling time), surface antigen expression, colony-forming units, osteogenic, and adipogenic differentiation ability, as well as Klotho expression and senescence-associated gene expression (IL1β, IL1α, IL6, IL8, TNFα, MCP-1, CCL4, CXCL2). Only few differences were observed in this study between the regenerative properties of younger and older BMA clot. In detail, younger BMA clot exhibited increased PDGF-AB synthesis and ACAN expression in comparison to older ones. However, these differences do not interfere with MSCs viability, proliferation, clone development, antigen expression, and osteogenic differentiation ability and with specific key GFs and genes related to aging (Ogino et al., 2006; Li et al., 2007; Tokunaga et al., 2008; Huang et al., 2009; Kumar et al., 2010; Wirz et al., 2011). We hypothesized that since PDGF-AB is the most abundant isoform of the PDGF family of GFs that is released upon stimulation with thrombin or other signals related to inflammation and injury by alpha granules in platelets, its lower expression in clotted BMA from older patients was due to the presence of an initial physiological lower platelet number in aging patients. In fact, a large clinical study that included more than 12,000 subjects, adjusted for many covariates such as nutritional deficiencies, medication, inflammatory conditions, autoimmune or viral illnesses, and consumption of alcohol and tobacco, indicated a significant decrease in 1 × 10^4^ platelets/μL in individuals in the 60–69 years age group as compared with those aged 20–59 years and in 2 × 10^4^ platelets/μL in patients aged over 69 years (Segal and Moliterno, 2006). Similar results were also observed in our study where the older individuals had an initial lower platelet number in comparison to younger individuals (214 ± 63.5 × 10^9^/L *versus* 266 ± 27.2 × 10^9^/L) (*data not shown*). Furthermore, our study showed that the production of PDGF-AB after 6 and 9 days of culture did not show significant differences between younger and older donors (*Supplementary Data*). Although the PDGF family of GFs is recognized as potent mitogens, chemoattractant, and survival factors for cells of mesenchymal origin, the relevance of PDGF-AB in stem cell proliferation and osteogenic differentiation is controversially discussed (Ogino et al., 2006; Li et al., 2007; Tokunaga et al., 2008; Huang et al., 2009; Wirz et al., 2011). It was reported that PDGF-AB binds to the receptor subtypes PDGF-Rαα and PDGF-Rαβ; however, it does not bind to PDGF-Rββ which is specifically associated with proliferation and preservation of stem cell function (Tokunaga et al., 2008; Kumar et al., 2010). However, other GFs such as TGF-β1, BMP2, FGF2, VEGF, and PDGF-CC did not show any significant differences between younger and older donors. In particular, TGF-β1 has anabolic and anti-inflammatory effects and regulates MSC proliferation and colonies formation. BMP2 is a potent osteoinductive molecule that induces osteogenic differentiation of responsive cells and stimulates bone healing and fracture repair. FGF2 enhances MSCs proliferation and cooperates in maintaining MSCs three-lineage differentiation potential during *in vitro* expansion. VEGF promotes angiogenesis and has a role in tissue healing. PDGF-CC is a well-established mitogen for MSCs (Enarsson et al., 2002; Ho et al., 2016; Grafe et al., 2018). In our study, a difference in MSCs’ ACAN expression was observed during chondrogenic differentiation. Both cultures, younger and older, differentiated towards the chondrogenic phenotype, but younger donors seem to differentiate more efficiently, regardless of ACAN. However, the expression of ACAN in pellet culture is not the only marker of chondrogenesis. Sox9, which promotes progression to chondrocyte differentiation at later stages, did not show significant differences (Akiyama et al., 2002; Ikeda et al., 2004; Lefebvre and Smits, 2005; Kozhemyakina et al., 2015; Liu and Lefebvre, 2015). Also, the gene expression of COL2A1, one of the core components of the extracellular matrix of articular cartilage, did not display changes between younger and older donors. Results of this study also indicated that both cultures, younger and older, were able to differentiate onto the adipogenic lineage, when exposed to definite inducing factors, without any significant differences. This result is of key interest for clotted BMA clinical application in aged patients. According to literature data, aging favors BM-MSCs adipogenesis at the expense of osteogenesis, with a consequent decrease in bone formation capacity, thus considerably reducing the clinical translation of BM-MSCs’ cell-based therapy in aged patients (Marędziak et al., 2016). This feature was not detected in our study where the adipogenic characteristics of MSCs from clotted BMA were not affected by age. These data were further strengthened by the absence of aging effects on the osteogenic potential. This process is regulated by RUNX2, the master regulator of osteogenic differentiation that in turn controls the expression of COL1A1 and BGLAP/osteocalcin. COL1A1 is an early osteoblast marker while BGLAP is a late differentiation marker expressed in the bone with a specific function associated with mineralization and matrix synthesis. These data, showing that the osteogenic differentiation potential of younger and older MSCs from clotted BMA is not affected by age, suggest that both cells’ sources could be considered equivalent for bone-related applications, emphasizing the advantages of these cells source for treating bone damage in the elderly populations. Although *in vivo* aging closely refers to donor age, *in vitro* aging is correlated not only to the loss of stem cells’ characteristics but also to the production of senescence-associated factors (Zhang Y et al., 2021). Thus, for MSCs to be clinically effective, it is also essential to monitor senescence-associated secretory factor and specific aging suppressor gene, such as Klotho gene. In our study, the expression level of IL1β, IL1α, IL6, IL8, TNFα, MCP-1, CCL4, and CXCL2 as well as Klotho produced by MSCs did not change between younger and older donors. These factors are crucial for MSCs’ senescence, but they are also able to self-regulate MSCs’ proliferation in culture (Zhang Y et al., 2021). In addition, senescence-associated secretory phenotype–mediated inflammatory factors are also interconnected with the inflammatory process. For example, in the presence of an inflammatory environment (e.g., high levels of TNFα, IL-6), MSCs become activated through toll-like receptor (TLR)4 and adopt an immune-suppressive phenotype by secreting high levels of soluble factors (Li et al., 2017). In this study, a substantial expression of IL-6 and MCP-1 was detected in both younger and older donors. These aspects, in association with the high concentration of GFs detected in this study, replicate what happens in the fracture hematoma microenvironment. After a fracture, coagulation and inflammation occur to create a matrix architecture, with the inflammatory cytokines that drive and enhance the coagulation cascades (Santos Duarte Lana et al., 2020). Thus, in our experimental scenario, the use of BMA clot seems to work as an early hematoma both in the younger and older population and therefore may be able to improve and enhance damaged tissues in an easy way without any sample processing and avoiding potential contamination risks as well as losing cell viability, proliferation, and differentiation ability.

In conclusion, this study demonstrated that donor age does not affect functional and phenotypical characteristics of clotted vertebral BMA that showed two successful hits working as a biological scaffold and as a powerful source of mesenchymal stem cells.

## Data Availability

The raw data supporting the conclusions of this article will be made available by the authors, without undue reservation.

## Glossary

BMA: bone marrow aspirate
MSCs: mesenchymal stem cells
GFs: growth factors
PDGF: platelet-derived growth factor
ACAN: Aggrecan
BM: bone marrow
BMC: BM concentrate
TGF-β: transforming growth factor beta
VEGF: vascular-endothelial growth factor
FGF2: fibroblast growth factor
SASP: senescence-associated secretory phenotype
IL: Interleukin
Runx2: runt-related transcription factor 2
COL1A1: collagen type I alpha 1
TRAP: tartrate-resistant alkaline phosphatase
MMP: metalloproteinase
CTSK: cathepsin K
HIV: human immunodeficiency virus
HBV: hepatitis B virus
HCV: hepatitis C virus
DMEM: Dulbecco’s modified Eagles medium
FBS: fetal bovine serum
PBS: phosphate-buffered saline
bFGF: basic fibroblast growth factor
BMP-2: bone morphogenetic protein 2
FCB: flow cytometry buffer
FITC: fluorescein isothiocyanate
IgG: immunoglobulin G
DT: population doubling time
CPDs: cumulative population doublings
PFA: paraformaldehyde
SD: standard deviation
